# FACS-Sortable Triple Emulsion Picoreactors for Screening Reactions in Biphasic Environments

**DOI:** 10.1002/admi.202400403

**Published:** 2024-12-04

**Authors:** Samuel Thompson, Yanrong Zhang, Zijian Yang, Lisa Nichols, Polly M. Fordyce

**Affiliations:** Department of Genetics, Stanford University, Stanford, CA 94305, USA; Stanford Shared FACS Facility, Stanford University, Stanford, CA 94305, USA; Department of Radiology, Stanford University, Stanford, CA 94305, USA; Center for Molecular and Genetic Medicine, Stanford University, Stanford, CA 94305, USA; Department of Genetics, Stanford University, Stanford, CA 94305, USA; Department of Bioengineering, Stanford University, Stanford, CA 94305, USA; Sarafan ChEM-H Institute, Stanford University, Stanford, CA 94305, USA; Chan Zuckerberg Biohub, San Francisco, CA 94158, USA

**Keywords:** biphasic reactions, catalyst, directed evolution, droplets, FACS, microfluidics, screening

## Abstract

Biphasic environments can enable successful chemical reactions where any single solvent results in poor substrate solubility or poor catalyst reactivity. For screening biphasic reactions at high throughput, a platform based on microfluidic double emulsions can use widely available FACS (Fluorescence Activated Cell Sorting) machines to screen millions of picoliter reactors in a few hours. However, encapsulating biphasic reactions within double emulsions to form FACS-sortable droplet picoreactors requires optimized solvent phases and surfactants to produce triple emulsion droplets that are stable over multi-hour assays and compatible with desired reaction conditions. This work demonstrates such FACS-sortable triple emulsion picoreactors with a fluorocarbon shell and biphasic octanol-in-water core. First, surfactants are screened to stabilize octanol-in-water emulsions for the picoreactor core. With these optimized conditions, stable triple emulsion picoreactors (>70% of droplets survived to 24 hr), produced protein in the biphasic core via cell-free protein synthesis are generated, and sorted these triple emulsions based on fluorescence using a commercial FACS sorter at >100 Hz with 75–80% of droplets recovered. Finally, an in-droplet lipase assay with a fluorogenic resorufin substrate that partitions into octanol is demonstrated. These triple emulsion picoreactors have the potential for future screening bead-encoded catalyst libraries, including enzymes such as lipases for biofuel production.

## 1. Introduction

Catalysts are essential to the modern chemical industry, and novel catalysts open up avenues to new medicines,^[1]^ materials with novel properties,^[2]^ and more sustainable production methods.^[3]^ To optimize reaction properties such as yield and enantiomeric excess, catalysts must be screened for compatibility and performance with each desired substrate. However, identifying an effective catalyst can be challenging when the substrate and catalyst are differentially soluble, as low effective substrate/catalyst concentrations and catalyst inactivation can limit product formation. Examples of such differentially soluble reactions include reactions with polar/hydrophilic catalysts and hydrophobic substrates with established applications for lipase-catalyzed biofuel production,^[4]^ biphasic variations of nitration reactions,^[5]^ and organometallic C—C bond formations via olefin metathesis^[ 6,7]^ or Suzuki–Miyura coupling.^[8]^

**Figure 1.**
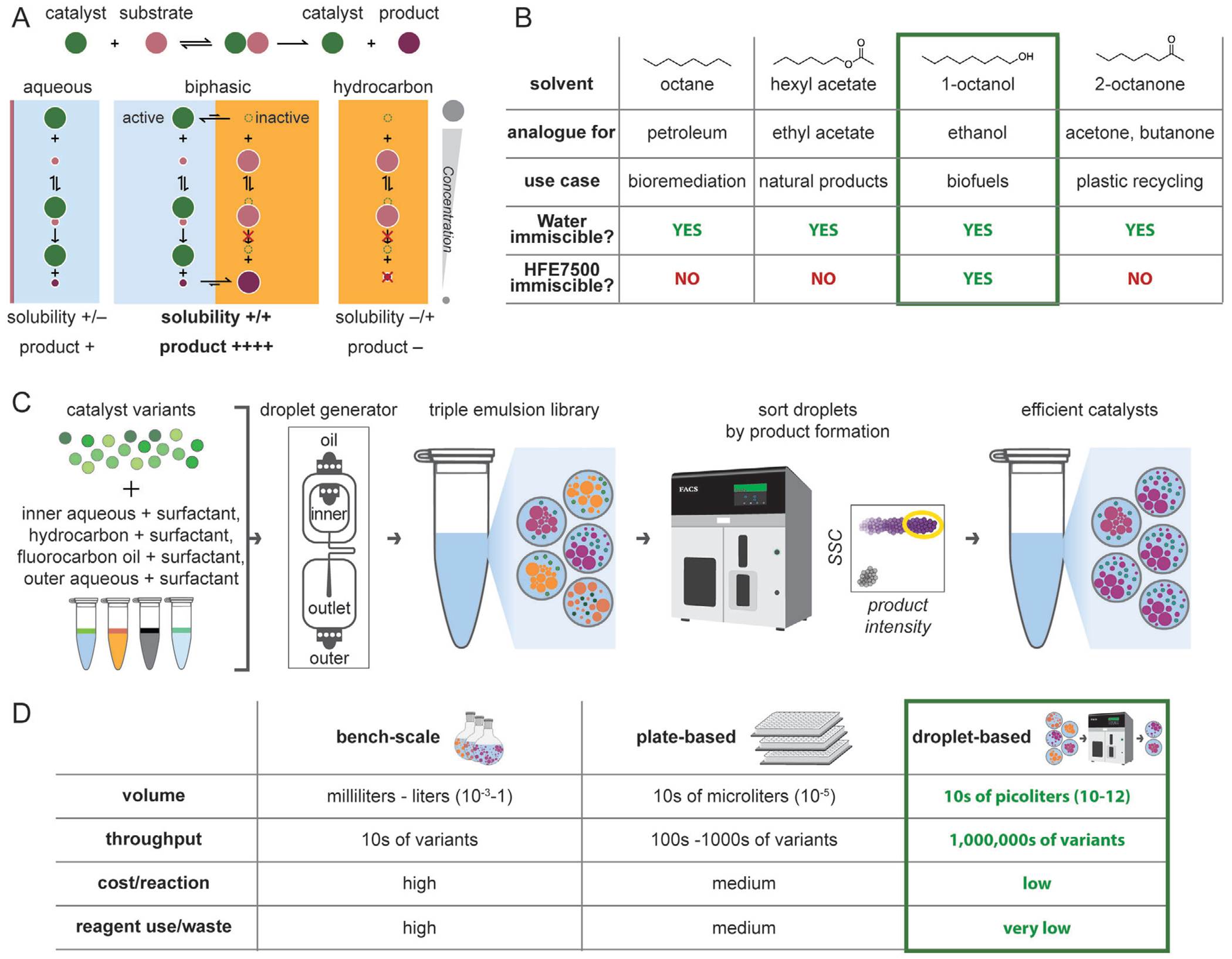
Triple emulsion picoreactors for optimizing biphasic reactions with wide-ranging applications. A) Schema illustrating how biphasic reactions use immiscible solvent systems to maintain high concentrations of substrate/product and active catalysts in separate phases. Rapid exchange between phases allows for turnover and accumulation of the product in the hydrocarbon phase. B) Example water-immiscible solvents. Solvents must be immiscible with both water and HFE7500 to be compatible with FACS (Fluorescence Activated Cell Sorting). Longer alkyl chains reduce solvent miscibility with water relative to more standard industrial solvents. C) Pipeline for screening biphasic reactions in FACS sortable triple emulsion picoreactors. D) Droplet microfluidics (green outline) increases throughput and reduces both cost and waste relative to traditional techniques.

This challenge can be circumvented using a biphasic reaction system with two immiscible phases (e.g., an aqueous phase containing most of the catalyst and a hydrocarbon phase containing most of the substrate and product) (Figure 1A). Despite catalyst and substrate preferring opposite solvent phases, substrate, product, and catalyst can still exchange between the two phases such that overall product formation rates depend on the rates of catalysis within each phase, the equilibrium concentrations, and kinetics of partitioning (Figure 1A). Hydrocarbon solvents with a variety of functional groups can form biphasic systems with polar and aqueous solvents, including alkanes, alcohols, ketones, and esters (Figure 1B). This wide variety of functional groups enables many useful processes in biphasic reactions, including petrochemical oxidation^[9]^ and bioremediation,^[10]^ plastic recycling,^[11,12]^ biofuel production,^[4,13]^ and natural product synthesis/extraction.^[14,15]^ Critically, the presence of an aqueous phase allows the application and benefits of enzymatic processes: 1) high activity at ambient temperature and pressure, 3) generally higher turnover, 3) biodegradability, and 4) production costs that can fall below $10 kg^−1^ of catalyst.^[16–18]^

Nevertheless, the optimization of biphasic reactions remains challenging. Beyond challenges common to all chemical reactions (e.g., yield, side product contamination, enantiomeric excess, etc.), optimizing biphasic reactions requires maximizing interfacial surface area over the duration of an experiment^[19]^ (e.g., via mixing^[20]^ or continuous and segmented flow in microfluidic reactors^[21–23]^) and identifying reaction-compatible solvents with low water-miscibility. Common hydrocarbon solvents contain few atoms and are often polar and miscible with water (e.g., fully: methanol, ethanol, acetone; partially: ethyl acetate). Solvents with the same polar functional groups and longer alkyl chains (e.g., hexyl acetate, 1-octanol, 2-octanone) have lower miscibility with water (Table S1, Supporting Information) but may retain desirable solvent properties (e.g., ketone solvation of plastic polymers^[24–26]^). While these more hydrophobic solvents have promise for potential use within biphasic systems, experimental pipelines capable of systematically testing and optimizing their use within high-throughput screening platforms have been lacking.

Microfluidics provides a particularly promising method for high-throughput screening of biphasic reactions, as the relatively small volumes and large interfacial surface areas can dramatically enhance small molecule transfer. Reflecting this, microfluidic slug/plug flow devices have been used to screen 10–100 biphasic reaction conditions at a time using in-line monitoring^[21,27,28]^ (Table S2, Supporting Information). Droplet microfluidics provides a potential way to scale these screens via high-throughput compartmentalization and sorting to recover and identify catalysts or conditions favorable for product formation. To date, single-phase droplet microfluidics has been used to screen for and isolate promising candidates from libraries of variants for organic,^[29]^ metallic,^[30]^ organometallic,^[31]^ and enzymatic catalysts^[32–35]^ via FADS (Fluorescence Activated Droplet Screening), MADS (Mass spectrometry Activated Droplet Screening), absorbance,^[36]^ or electrochemistry,^[37]^ all of which require custom device fabrication and sorting equipment. More recently, double emulsion droplets have enabled ultra-high-throughput encapsulation, screening, and isolation of pL-volume reactions using only simple microfluidic devices and commercially-available equipment.^[38–41]^ In double emulsion droplets, reactions of interest are encapsulated within a thin fluorocarbon oil shell such that they can be loaded into and sorted by commercially available FACS (Fluorescence Activated Cell Sorting) instruments at rates of up to 1–5 kHz (9 million droplets/hour).

Double emulsion droplet screening platforms compatible with biphasic reactions – triple emulsions – would allow for efficient search through complex combinatorial chemical spaces for desirable catalysts (e.g., DNA-encoded small molecule catalysts or directed evolution of enzymes that can be generated via cell-free synthesis). However, the lack of methods for generating stable FACS-sortable triple emulsion picoreactors is a significant technical barrier to realizing such a high-throughput screening platform. This method development requires (1) selecting 3 mutually immiscible phases (aqueous, hydrocarbon, and fluorocarbon) (Figure 1B; Figures S1, S2, Supporting Information) optimizing surfactants to stabilize those interfaces, (3) producing triple emulsion picoreactors, (4) characterizing triple emulsion stability, and (5) confirming triple emulsion compatibility with both the desired reaction and flow cytometry/cell sorting instruments.

Here, we present the generation of novel triple emulsion picoreactors that encapsulate a biphasic reaction environment and have been optimized for high-throughput screening using FACS. Hydrocarbon/aqueous biphasic solutions are encapsulated within fluorocarbon oil shells to form triple emulsions that can be screened using commercially available FACS instruments, with applications to high-throughput screening of enzyme variants produced via cell-free protein synthesis (Figure 1C). To achieve this, we first developed and deployed a novel plate-based screening pipeline to identify combinations of solvents and surfactants capable of forming stable hydrocarbon/aqueous emulsions (e.g., octanol with aqueous buffer). We then loaded stable hydrocarbon/aqueous emulsions into droplet generators to yield triple emulsion picoreactors (Figure S1, Supporting Information) that remained stable over tens of hours. We demonstrated cell-free protein synthesis within the aqueous phase of triple emulsion picoreactors and sorting of triple emulsion picoreactors without custom equipment, which should enable library screens with >10^6^ variants while drastically reducing the reagent volumes required (Figure 1D).

## 2. Results

### 2.1. An Optimized Experimental Pipeline to Identify Hydrocarbon + Aqueous + Surfactant Combinations

**Figure 2.**
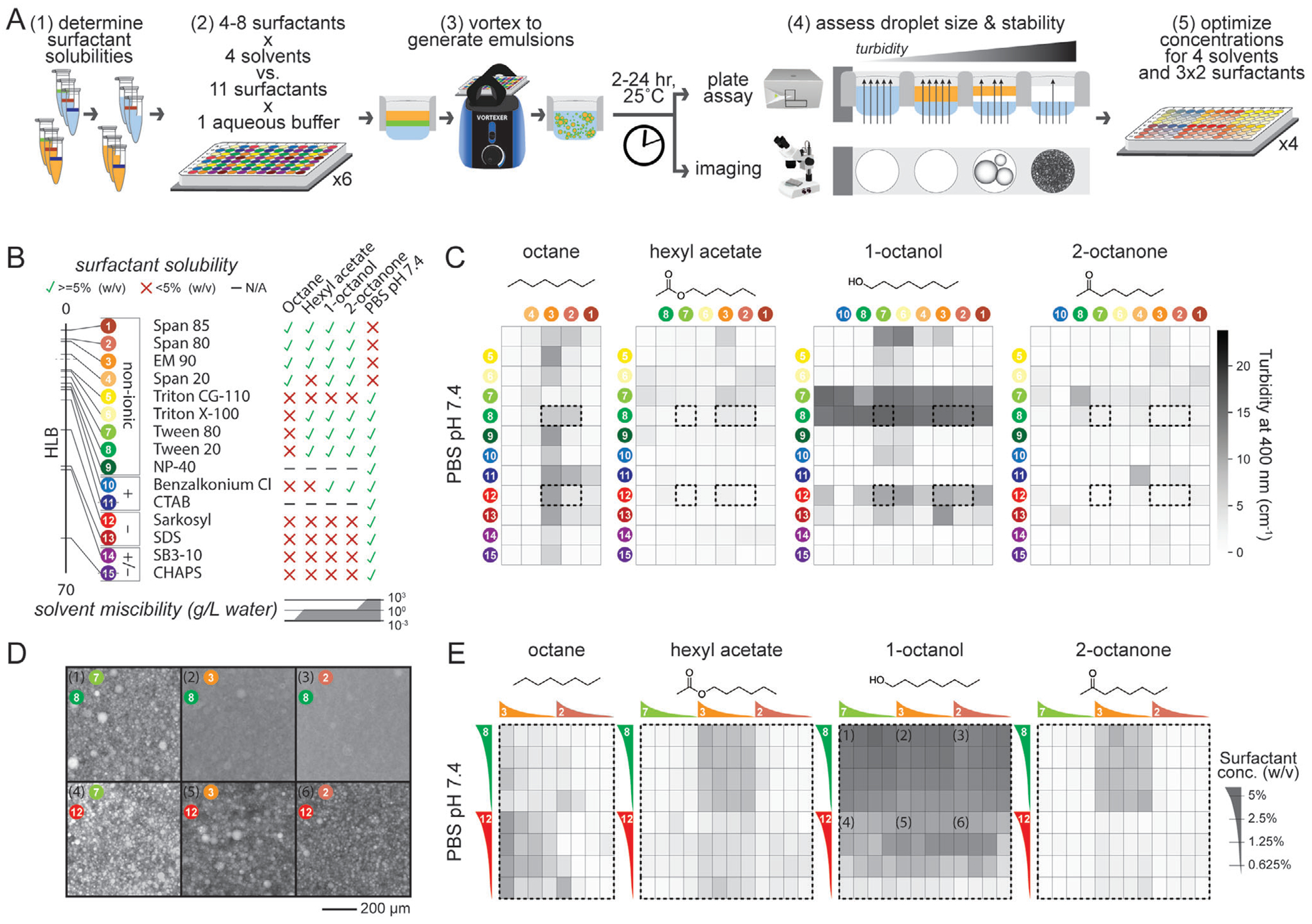
Turbidity- and microscopy-based screen identifies surfactants for stabilizing hydrocarbon/aqueous single emulsions. A) Workflow for rapid assay to screen optimal surfactant conditions by 1) testing if surfactants are soluble in aqueous buffer and/or hydrocarbon solvents, 2) selecting a surfactant panel based on the number of soluble surfactants for each solvent, 3) vortexing to form emulsions, 4) screening for droplet formation in a multimode plate reader and microscopy, and 5) optimizing concentrations for droplet size and stability. B) Solubility for selected surfactants arranged by hydrophilic-lipophilic balance (HLB) and categorized by charge as non-ionic, cationic (+), anionic (−), or zwitterionic (+/−). Green checks and red Xs indicate solubility at > or < 5% (w/v), respectively. C) Plate reader turbidity measurements after 24 h for one aqueous buffer (PBS pH 7.4) and 4 hydrocarbon solvents with surfactant combinations. Dashed black outlines indicate selected conditions used in D and E. D) Fluorescence microscopy images of octanol/aqueous emulsions stabilized by the selected surfactants from C); octanol is fluorescently labeled with Nile Red. Scale bar: 200 μm. E) Turbidity measurements as in C from screening selected surfactants over concentrations from 0.625 to 5% (w/v).

Successful FACS-based screening of biphasic solvent/water reactions requires: 1) that hydrocarbon-in-water droplets can be encapsulated within a fluorocarbon shell (Figures S1, S2, Supporting Information), 2) that these triple emulsions remain stable over time, and 3) that the overall dimensions of the triple emulsion are sufficiently small to maximize interfacial surface area and pass through FACS nozzles without disrupting stable water-in-air droplet breakoff. Maintaining triple emulsion stability depends critically on the use of surfactants to tune interfacial surface tensions at each interface (inner hydrocarbon/aqueous, inner aqueous/oil, and oil/outer aqueous, see Figure S1, Supporting Information). Consistent with this, initial naïve attempts to generate triple emulsions without surfactant in the hydrocarbon/aqueous biphasic solution destabilized the fluorocarbon oil shells and caused jetting, preventing successful droplet generation and sorting (Figure S3, Supporting Information). To systematically and efficiently identify promising hydrocarbon + aqueous + surfactant combinations, we leveraged the fact that changes in the number and size of emulsified droplets for two fluids with different refractive indices alter light transmission. Thus, optical properties (e.g., optical density, absorbance, turbidity) can be used to monitor emulsion stabilities.^[42,43]^ Specifically, we: 1) tested surfactant solubilities in aqueous buffer and hydrocarbon solvents, 2) vortexed mixtures of aqueous buffer, hydrocarbon solvents, and aqueous- and hydrocarbon-compatible surfactants to emulsify them, and then 3) assessed droplet formation and stability via plate-based light transmission assays and microscopy; after identifying promising combinations, we performed an additional screen to optimize surfactant concentrations (Figure 2A). While we focus here on octanol because of its compatibility with fluorinated oils required to create FACS-sortable microfluidic droplets, this general approach could be used to optimize reaction conditions for a wide range of alternative screens.

As a first step in screening for promising fluid/surfactant combinations, we assessed the solubility of 15 readily available commercial surfactants in a standard aqueous buffer (phosphate buffered saline (PBS)) pH 7.4, a model enzyme reaction buffer) and four 8-carbon solvents (octanol, octane, 2-octanone, and hexyl acetate) (Figure 2B; Table S1, Supporting Information). All 4 solvents are known to form a biphasic system with water, with reported solubilities in water of 0.00066 g L^−1^ for octane and 0.3–0.9 g L^−1^ for the remaining solvents. The 15 surfactants included 9 non-ionic surfactants, 2 cationic surfactants, 2 anionic surfactants, and 2 zwitterionic surfactants spanning a broad range of hydrophilic-lipophilic balance (HLB) values (see Methods for further discussion). Nearly all ionic surfactants (CHAPS (3-((3-cholamidopropyl) dimethylamino)-1-propanesulfonate), SB3-10, Sarkosyl, SDS (sodium dodecyl sulfate)) were soluble only in PBS pH 7.4, except for benzalkonium chloride (which was also soluble in 1-octanol and 2-octanone); two surfactants were provided as aqueous solutions (NP-10, CTAB (cetrimonium bromide)), precluding solvent solubility tests. While surfactants with the lowest HLB values (e.g., EM-90, Span) were soluble only in organic solvents (Table S3, Supporting Information), other non-ionic surfactants were generally soluble in PBS pH 7.4 (e.g., Triton X-100, Tween-80, and Tween-20). While solubility generally trended with surfactant HLB values and solvent miscibility values (Figure 2B; Table S3, Figure S4, Supporting Information), the relationship was not fully predictive (e.g., for Triton CG110 and for Span 20), establishing a need for direct empirical testing.

### 2.2. Plate-Based Turbidity Assay Can Screen For Surfactants That Stabilize Hydrocarbon/Aqueous Emulsions

Next, we tested all possible combinations of aqueous buffer (1), hydrocarbon solvent (4), aqueous-soluble surfactant (11), and hydrocarbon-soluble surfactants (8) for their ability to form emulsions (8 plates in total) by including each surfactant at a single high concentration (5% w/v) and quantifying emulsion stability at 2 and 24 h via a plate-based turbidity screen and microscopy (see Supporting Information). Plate-based screening revealed differences in turbidities as a function of surfactant conditions and over time, providing measurements of droplet formation and stability (Figure 2C; Figures S5–S11, Supporting Information). Fine emulsions formed for each solvent, with some wells appearing visibly white and opaque (in contrast to the optically clear appearance of conditions without surfactant). After 2 h, maximum turbidities for each solvent ranged from 10.6 to 19.3 cm^−1^, consistent with optically dense solutions of fine emulsions; a higher fraction of octane conditions formed fine, optically dense emulsions. After 24 h, median turbidity values dropped but maximum turbidities remained high, consistent with a general demulsification across conditions but with the finest emulsions being most stable. As expected, turbidity values at 24 h negatively correlated with mean intensities of microscopy images (Figure S12, Supporting Information); hexyl acetate and 2-octanone emulsions clustered at well edges (Figures S9, S11, Supporting Information) but octanol emulsions appeared well-dispersed (Figure S10, Supporting Information). Overall, 24 surfactant conditions with octanol were somewhat stable (turbidity >9.8 (50% of maximum) after 24 h); 11 conditions were highly stable (turbidity >17.6 (90% of maximum)) and frequently contained Tween, Span, and EM90 surfactants. To more carefully examine the impacts of surfactants on emulsion formation and stability, we regenerated octanol/aqueous emulsions for a subset of surfactants that produce highly stable emulsions in octanol and produce emulsions with a wide range of stabilities over all four hydrocarbon solvents (Tween 80, EM90, and Span 80 hydrocarbon solvent surfactants; Tween 20 and Sarkosyl aqueous surfactants) and imaged the resulting emulsions via microscopy (Figure 2D; Figure S13, Supporting Information). High turbidity (Figure 2C) provided information about emulsion droplet size; changes in turbidity (Figure S6, Supporting Information) provided a convenient metric of emulsion stability. Light microscopy analysis of emulsion samples in chamber slides confirmed that Tween 20 and Sarkosyl stabilize octanol/aqueous emulsions with μm-scale droplets. Tween 20 yields slightly finer emulsions, consistent with higher measured turbidities. Droplets generated with Tween 20 had smaller oil droplet radii (median = 1.0–1.75 μm) than those generated with Sarkosyl (median = 2.7–4.5) (Figure S14, Supporting Information), making them more compatible with loading into double emulsions (core radius: 10–15 μm).

To assess the concentration-dependence of surfactant stabilization, we repeated screens with surfactant concentrations from 0.625–5% (Figure 2E; Figures S15–S21, Supporting Information). Droplet stabilities (minimally changing turbidity values) generally increased with increasing Sarkosyl in the aqueous phase regardless of octanol surfactant concentrations while Tween-20 impacts were more concentration-independent (Figure S16, Supporting Information). These results establish that increasing surfactant concentration does not always increase emulsion stability and again highlight a need for direct experimental screening. Ultimately, we identified many surfactant conditions that can stabilize micron-scale hydrocarbon/aqueous emulsions for tens of hours, providing a starting point for further optimization to specific assays and applications.

### 2.3. Triple Emulsion Picoreactors Can be Formed by Encapsulating Octanol/Aqueous Emulsions in Fluorocarbon Oil Shells

**Figure 3.**
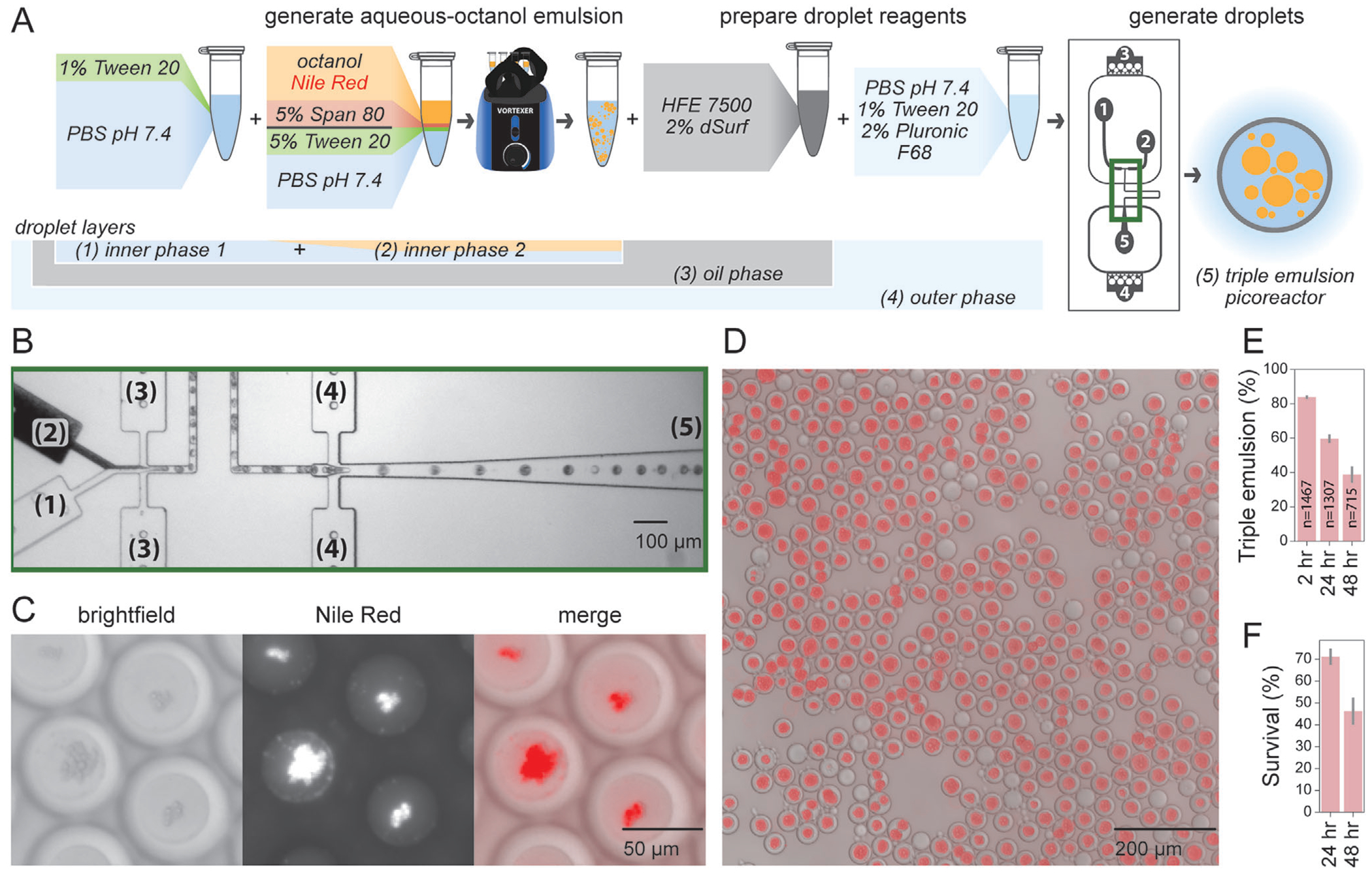
Encapsulating hydrocarbon/aqueous single emulsions in double emulsions produces triple emulsion picoreactors. A) Workflow for generating triple emulsions by encapsulating pre-emulsified octanol/aqueous emulsions in the core of aqueous/fluorocarbon/aqueous double emulsions. Numbered inputs and outputs match the ports/channel on the droplet generator schematic. B) Microscopy images of droplet generation at the region of the droplet generator outlined in green in A. Channels numbered as in A. C) Brightfield and fluorescence microscopy images of triple emulsions; octanol is fluorescently labeled with Nile Red. D) Merged brightfield and fluorescence image of produced triple emulsion droplets. E) Percentage of triple emulsions within produced droplet population over time. Error bars represent standard deviation in percentages across 3 images. Number of total droplets indicated on each bar. F) Survival rates reported as the ratio of triple emulsion percentages at 24 h versus at 2 h.

After identifying multiple reagent combinations capable of forming stable octanol/aqueous emulsions, we next attempted to generate triple emulsion droplets using an octanol/aqueous/fluorocarbon solvent system because all three phases are mutually immiscible. We tested whether emulsions containing these surfactants could be successfully and stably encapsulated within an oil shell to yield FACS-sortable triple emulsions (Figure 3A). Specifically, we: 1) labeled octanol with Nile Red, a lipophilic dye that preferentially partitions into octanol rather than aqueous buffer or fluorinated oil (Figure S22, Supporting Information), 2) generated octanol/aqueous droplets with relatively small median radii via vortex emulsification using Tween-20 and Span-80 as the aqueous and octanol surfactants, respectively (Figure 2D; Figure S13, Supporting Information), 3) introduced this octanol/aqueous emulsion as the inner aqueous phase within a double emulsion droplet generator (Figure 3B), and then 4) collected and imaged the resultant output droplets via brightfield and fluorescence microscopy (see Supporting Information). The octanol/aqueous emulsion appeared opaque within the droplet generator inlets and yielded droplets comprised of an oil shell and an opaque core, many of which contained one or more smaller inner droplets. As expected, the lipophilic Nile Red dye colocalized with the small droplets within the inner core, confirming the successful formation of FACS-sortable triple emulsion droplets (Figure 3C,D). Overall, more than 80% of droplets displayed the desired triple emulsion architecture (83.9 ± 1.1%; n = 1467), with a mean triple emulsion radius of 32.0 ± 4.1 μm (Figure 3E; Figures S23, S24, Supporting Information). Triple emulsions were stable over tens of hours, with 71.1 ± 3.8% of droplets surviving to 24 h (Figure 3E). These rates establish the ability to generate droplets populations consisting primarily of triple emulsions.

### 2.4. Proteins Can be Expressed In Situ Within Triple Emulsion Picoreactors

**Figure 4.**
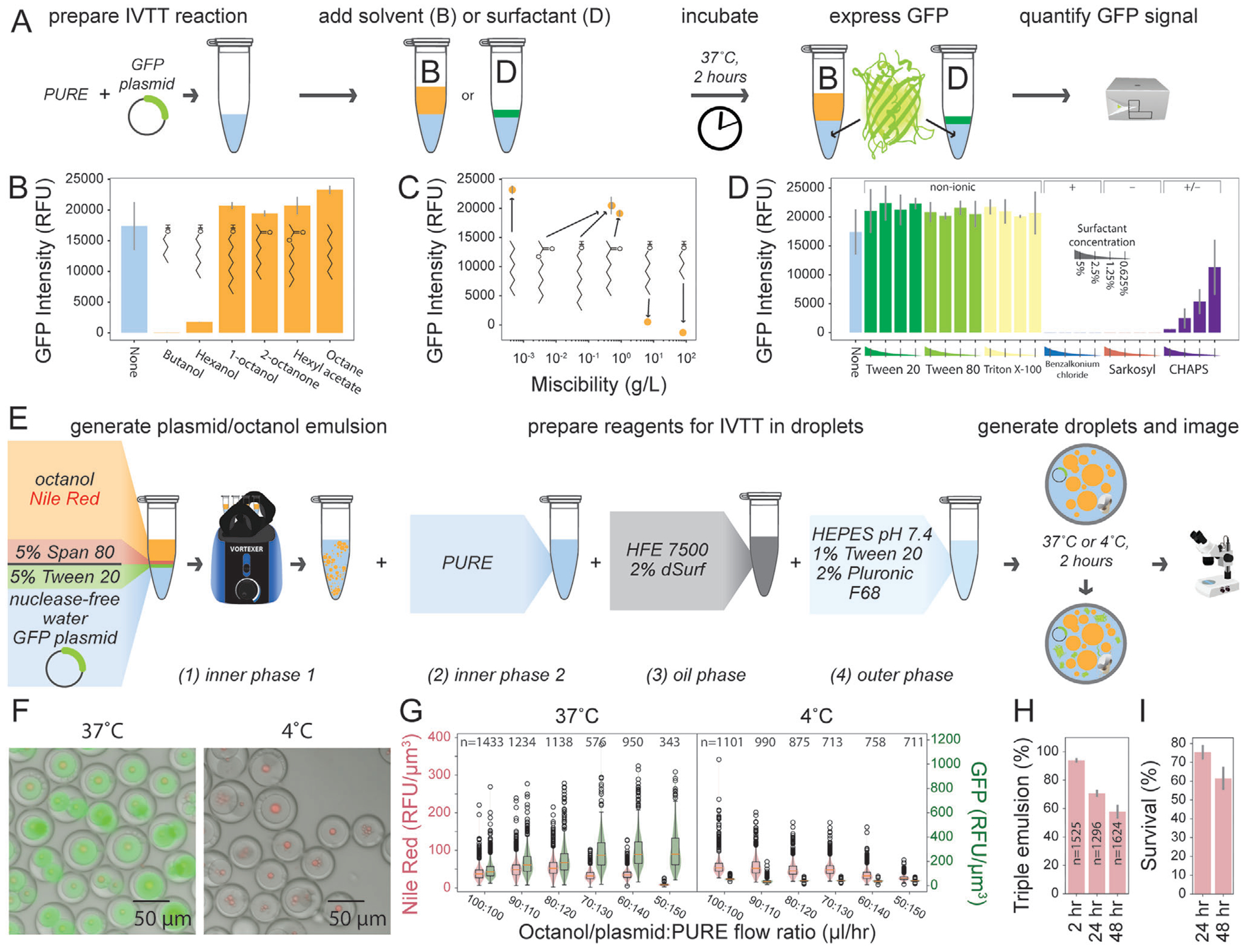
In vitro transcription translation yields expressed protein within triple emulsion picoreactors. A) Workflow for testing IVTT compatibility with aqueous-hydrocarbon emulsions. GFP expression with PURE reagents in the presence of solvent (B) or surfactant (D) quantifies transcription/translation activity in solution. B) GFP signal for IVTT reaction in the presence of equal volumes of hydrocarbon solvent. Error bar represents standard deviation from measurements of 3 reactions. C) GFP signal from B plotted against solvent miscibility in water. D) GFP signal for IVTT reaction with 0.625–5% water-soluble surfactant. Error bar represents standard deviation from measurements of 3 reactions. Labels indicate surfactant charge. E) Workflow for expressing GFP in triple emulsion picoreactors. Protein expression is performed at 37 °C (positive control) or 4 °C (negative control); octanol is fluorescently labeled with Nile Red. F) Merged images of triple emulsion picoreactors with IVTT reagents incubated at 37 and 4 °C showing brightfield, GFP fluorescence (produced protein), and Nile Red fluorescence (octanol droplets). G) Nile Red and GFP fluorescence intensities across various flow rate ratios for octanol/plasmid + PURE reaction mixture. H) Percent triple emulsions within the output droplet population. Error bars represent the standard deviation across 3 images. I) Survival rates reported as the ratio of triple emulsion percentages at 24 and 48 h versus at 2 h.

Enzyme catalysts are particularly amenable to high-throughput screening strategies when produced via in vitro cell-free protein synthesis within an aqueous phase.^[44,45]^ To test whether our triple emulsion picoreactors can be used to express and screen enzyme catalysts, we first tested the compatibility of in vitro transcription/translation (IVTT) with reagents required to form and stabilize droplets (Figure 4A). To identify solvents compatible with IVTT, we combined a plasmid encoding expression of green fluorescent protein (GFP) with PURE (Protein expression Using Recombinant Elements) reagents and hydrocarbon solvents at a 1:1 ratio, incubated to allow for protein expression, and then quantified GFP fluorescence (Figure 4B). All four 8-carbon solvents supported expression (1-octanol, 2-octanone, hexyl acetate, and octane), with GFP signal negatively correlated with solvent miscibility in water (Figure 4B,C; Table S2, Supporting Information). Next, we investigated which water-soluble surfactants were compatible with IVTT by quantifying fluorescence intensity after expressing GFP in PURE reactions containing 0.625–5% of water-soluble surfactants (Figure 4D). While non-ionic surfactants (Tween, Triton) had no impact on GFP expression, cationic and anionic surfactants (benzalkonium chloride, sarkosyl) completely ablated expression; the zwitterionic surfactant CHAPS reduced GFP signal in a concentration-dependent manner (Figure 4D). Next, we tested for the ability to successfully produce proteins within triple emulsions for high-throughput screening by: 1) generating triple emulsion droplets with inner cores comprised of octanol/aqueous droplets containing Nile Red dye, Tween 20 and Span 80 surfactants, plasmid DNA encoding GFP expression, and all reagents required for IVTT, 2) incubating to allow IVTT and GFP production, and then 3) imaging to quantify the amount of expressed GFP in each droplet (Figure 4E, Supporting Information). To eliminate the possibility of GFP expression prior to droplet formation, inner core reagents were introduced separately via 2 inputs such that the plasmid DNA and IVTT reagents did not contact each other until just before droplet formation within the device. Droplets incubated at 37 °C for 2 h showed strong fluorescence in the green channel while negative control droplets incubated at 4 °C (a temperature below that required for IVTT) did not fluoresce (Figure 4F). Varying flow rates for the plasmid-octanol solution and the IVTT reagents (thereby changing the relative volume fractions of each solution within the inner core) led to concomitant changes in Nile Red and GFP intensities, consistent with either IVTT reagents being the limiting factor for expression in this experiment or an inhibitory impact of octanol when present at very high surface areas (Figure 4G). For the 100:100 flow ratio condition, the output droplets consisted of 94.0 ± 1.5% (n = 1525) triple emulsions with a mean triple emulsion radius of 35.0 ± 4.8 μm (Figure 4H; Figure S25, Supporting Information). About 75.3 ± 3.8% of triple emulsions incubated for 2 h at 37 °C survived to 24 h at room temperature and 61.5 ± 6.1% survived to 48 h (Figure 4I). These results indicate that biocatalysis can be performed in triple emulsion picoreactors, including for challenging reactions that require tens of hours.

### 2.5. Desired Picoreactor Populations Can be Selected and Recovered via FACS Sorting

**Figure 5.**
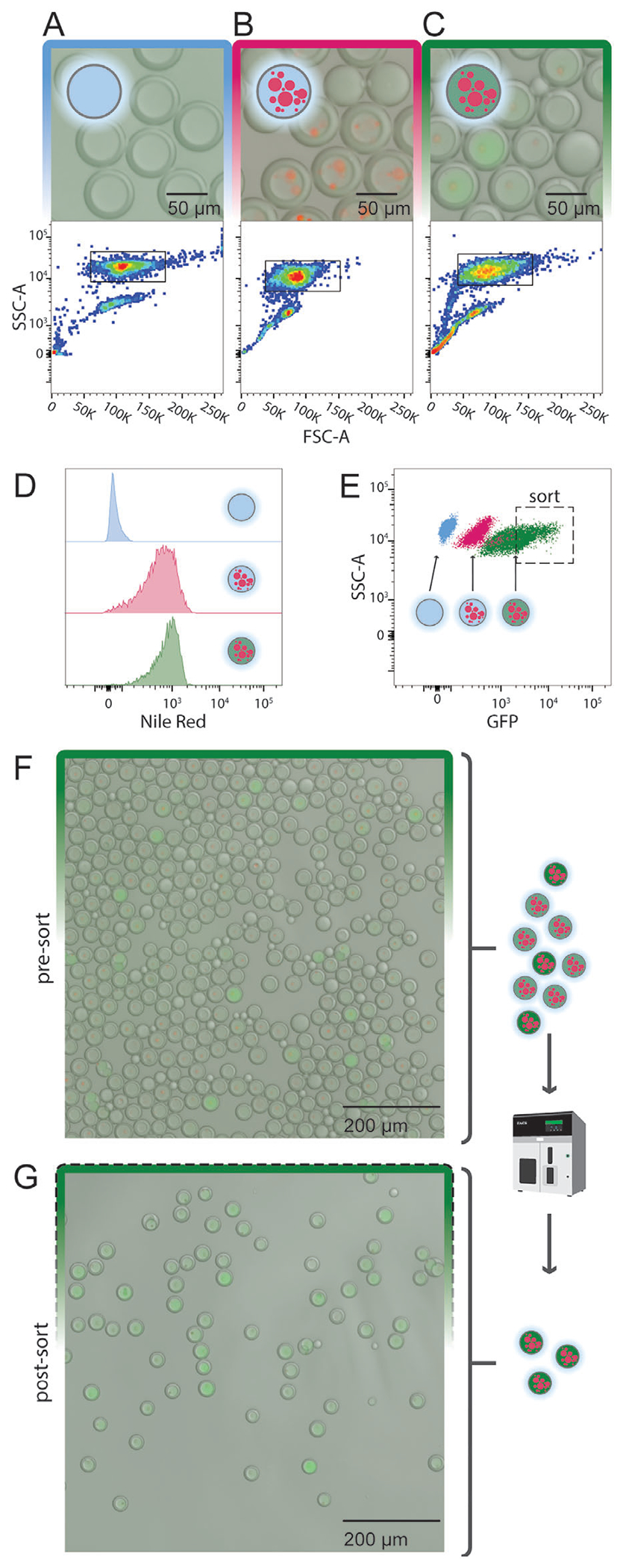
FACS sorting recovers target picoreactor subpopulation. Presort images (top) and side scatter versus forward scatter FACS distributions (bottom) for A) double emulsions, B) triple emulsion picorectors incubated at 4 °C to prevent protein expression. or C) incubated at 37 °C to promote protein expression. Black outline indicates FACS droplet quality gates. D) Histograms of Nile Red FACS signals for each sample. E) GFP FACS signals versus side scatter for each sample. Dashed black outline indicates FACS sorting gates. F) Images of triple emulsion picoreactors incubated at 37 °C before and G) after sorting.

Next, we tested if our triple emulsion picoreactors could be analyzed and sorted via FACS. To increase the recovery of sorted droplets, we reduced the size of the droplets by scaling the dimensions of the microfluidic devices to 2/3. With these smaller droplet generators, we used the optimized surfactants described above to produce triple emulsions containing IVTT reagents and Nile Red-labeled octanol and double emulsions that contained no fluorophore (Figure 5A–C). The triple emulsion output contained 90.9 ± 1.8% (n = 1154) triple emulsions with a mean triple emulsion radius of 26.4 ± 2.6 μm, and the double emulsion output contained >99.0 ± 0.0% (n = 506) double emulsions with a mean double emulsion radius of 29.4 ± 1.9 μm (Figures S26, S27, Supporting Information). We incubated the triple emulsions at 4 °C (to inhibit GFP expression) or 37 °C (to promote GFP expression) for 2 h and sorted the separate droplet populations by FACS at event rates of 100–800 Hz. The target droplet population comprised 83.0% (double emulsions), 56.2% (triple emulsions incubated at 4 °C), and 82.6% (triple emulsions incubated at 37 °C) of all events, respectively (Table S4, Supporting Information). Double and triple emulsion populations were clearly visible on plots of FSC-A (Foward Scatter - Area) versus SSC-A (Side Scatter - Area) (Figure 5A–C). As expected, double emulsions showed low signal for Nile Red (indicating an absence of octanol) and both triple emulsion samples showed increased and comparable Nile Red signal, confirming that triple emulsions can be reliably identified by fluorescence (Figure 5D). Consistent with microscopy results, signal intensity was ≈4x higher for triple emulsions incubated at 37 °C (median = 1595.9) than for triple emulsions incubated at 4 °C (median = 390.3) and double emulsions (median = 76.2) (Figure 5E). After sorting the triple emulsions incubated at 37 °C to select for droplets with the highest GFP signal (Figure S28, Table S4, Supporting Information), recovered droplets contained 96.0 ± 3.1% (n = 703) triple emulsions and were enriched for high fluorescence droplets (Figure 5F,G; Figure S26, Supporting Information), with 75–80% recovery (see Supporting Information). These results confirm that triple emulsion picoreactors can be sorted by fluorescence and recovered for downstream assays.

### 2.6. Enzyme Turnover in a Biphasic Reaction Can be Detected in Triple Emulsions

**Figure 6.**
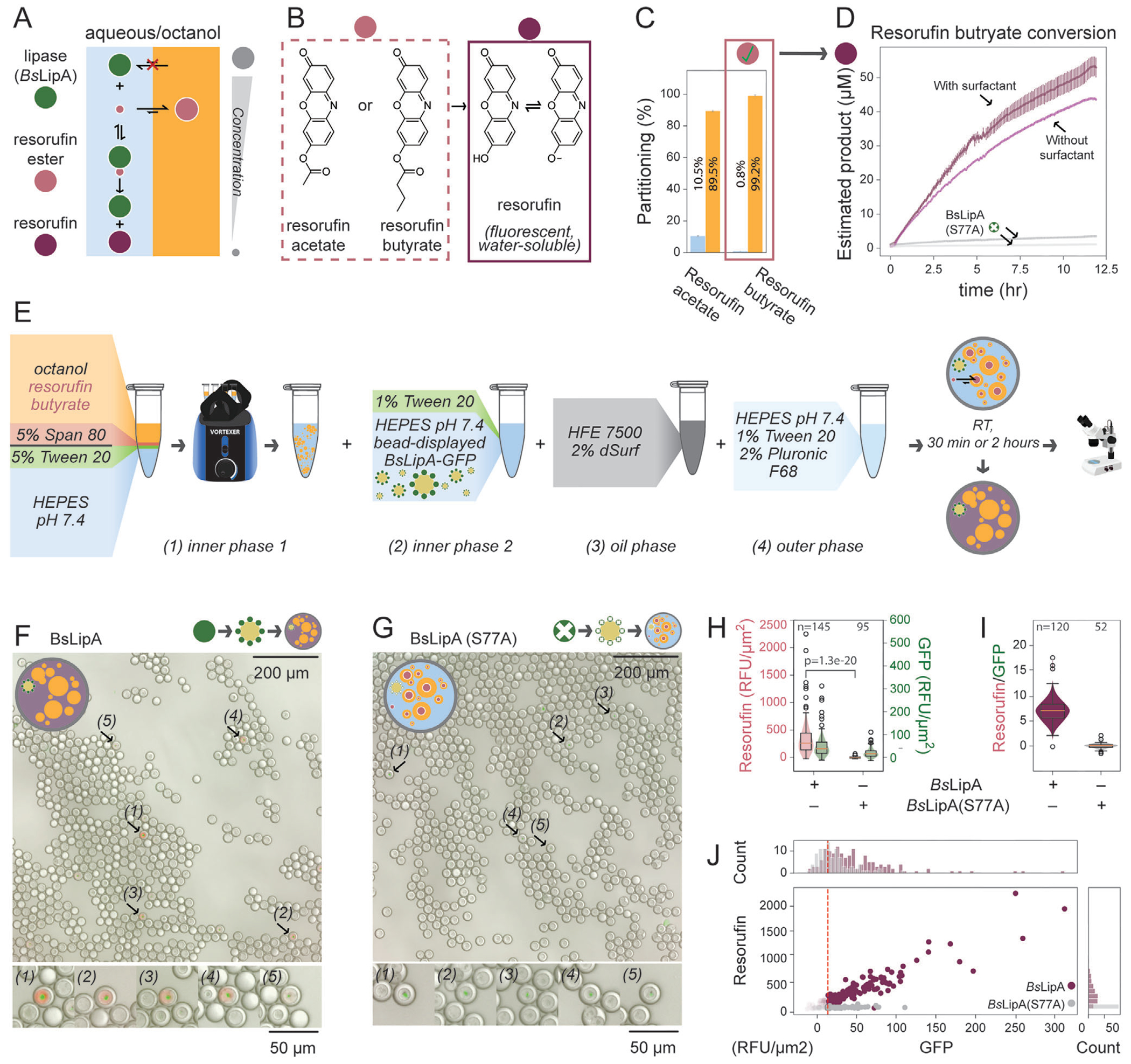
A lipase turns over an octanol-partitioning substrate within triple emulsion picoreactors. A) Schema illustrating turnover of resorufin esters that partition predominantly into the octanol phase by an enzyme in the aqueous phase. B) Two resorufin ester substrates (light purple) converted to a fluorescent resorufin product (dark purple) by the esterase activity of a lipase. C) Measured partitioning between aqueous buffer (blue) and 1-octanol (yellow) for two resorufin esters. Error bar represents standard deviation from measurements of 3 extraction samples. D) Measured resorufin butyrate turnover by BsLipA (purple) or BsLipa(S77A) (grey) in a biphasic reaction with (dark colors) or without (light colors) surfactants. Error bar represents standard deviation from measurements of 3 reactions. E) Workflow for assaying lipase activity in triple emulsion picoreactors. Turnover in droplets is initiated at formation by delivering GFP capture beads bound with eGFP-tagged *Bs*LipA (positive control) or BsLipA(S77A) (negative control); octanol contains the resorufin butyrate substrate. F,G) Merged brightfield, green fluorescence (eGFP), and red fluorescence (resorufin) images of triple emusion picoreactors with bead-displayed (F) BsLipA or (G) BsLipA(S77A). Arrows indicate numbered particles containing beads shown in the inset images below. H) Resorufin and eGFP fluorescence intensities across droplets with bead-bound BsLipA or BsLipa(S77A) after a 2 h incubation. The P-value for comparing the resorufin signal between two populations was calculated from a Student’s T-test. I) Comparison of the ratio of Resorufin to eGFP fluorescence intensities for all droplets above a noise threshold for eGFP signal (see Extended Methods). J) Comparison of resorufin and eGFP fluorescence intensities for the bead-containing droplet populations in H. The eGFP noise threshold is indicated with a dashed red line. Colors are shown as in panel (I) with points below the eGFP noise threshold are shown at 5% opacity.

Finally, we tested if enzyme activity in a biphasic reaction is compatible with these triple emulsions. In this biphasic reaction, the enzyme remains in the aqueous phase and processes a substrate that makes rare excursions from a hydrophobic phase (Figure 6A). We focused on lipases as a potential model system based on their industrial relevance and the availability of fluorogenic substrates we predicted would primarily partition into octanol. Lipases cleave ester bonds and have commercial applications in detergents, stereoselective synthesis methods, and biofuel synthesis.^[4,46,47]^ To test the compatibility of lipase assays with triple emulsions, we attempted to measure turnover for an engineered variant of Lipase A from *B. subtilis* (*Bs*LipA)^[48,49]^ interacting with an esterified fluorophore (e.g., resorufin) that mimics the quenched protonated state of the free fluorophore and is deprotected by nucleophilic activity (Figure 6B). While the deprotonated fluorophore product accumulates in the aqueous phase (pKa 6.0), esterified forms primarily partition into the octanol phase with the degree of partitioning varying with the length of the alkyl chain (Figure 6C; Figure S29, Supporting Information).

To test the ability to measure lipase activity within a biphasic reaction, we expressed WT *Bs*LipA and a catalytically dead variant (S77A) in PURE as SNAP-*Bs*LipA-eGFP fusions and incubated with 100 μM resorufin butyrate in octanol/water biphasic reaction environment without surfactants. Substantial product turnover was observed for the WT *Bs*LipA but not for the S77A mutant, confirming enzyme-mediated catalysis (Figure 6D). In contrast, aqueous-only reactions showed high background turnover from solvolysis (Figure S30, Supporting Information). Turnover was also faster in the aqueous phase, indicating that this model biphasic reaction is rate limited by the kinetic partitioning of the substrate into the aqueous phase. The addition of emulsion-stabilizing surfactants (5% Tween 20 in the aqueous phase and 5% Span 80 in the octanol phase) slightly increased turnover, consistent with a model in which increased surface area from emulsification promotes enhanced kinetic partitioning.

Next, we tested if this biphasic reaction could take place in triple emulsion droplets with bead-bound BsLipA-eGFP and resorufin substrate in the octanol phase. Specifically, we generated triple emulsions with an inner aqueous/octanol emulsion comprised of: 1) SNAP-*Bs*LipA-eGFP or SNAP-*Bs*LipA (S77A)-eGFP bound to 2.8 μm polystyrene magnetic beads displaying anti-eGFP antibodies in HEPES buffer, 2) resorufin butyrate in the octanol phase, and 3) Tween 20 and Span 80 surfactants. Bead concentrations were deliberately low such that most droplets were empty, whereas bead-containing droplets often incorporated visible bead clumps. Thus, the effective concentration of enzyme in each droplet varies. To eliminate the possibility of turnover prior to droplet formation, inner core reagents were introduced separately via two inputs such that the bead-displayed lipase and resorufin butyrate did not contact each other until just before droplet formation within the device. After incubating for 2 h to allow enzymatic turnover, imaging revealed fluorescence signals in bead-containing triple emulsions droplets containing WT *Bs*LipA with diffuse resorufin intensities linearly correlated with localized bead-associated eGFP intensities (Figure 6E–J, Supporting Information). By contrast, triple emulsion droplets containing the *Bs*LipA S77A mutant showed only eGFP fluorescence without significant resorufin signal (p = 1.3 × 10^−20^). Overall, these results demonstrate that enzymes can be active in triple emulsion droplets, and turnover can be detected with a fluorescence readout hours after droplet generation.

## 3. Discussion

For new catalysts in general, much attention has been directed to developing large-scale screens for evaluating catalysts for aqueous reactions^[30]^ and using machine learning algorithms to predict likely candidates from prior data.^[50,51]^ Thus, there is a natural synergy between high-throughput screening methods and state-of-the-art data analysis. Droplet microfluidic technologies have great promise for screening complex reaction conditions where the optimization space is large such as combinatorial condition space of biphasic reactions versus the chemical space of small molecule catalysts or the sequence space of enzymes using bead-display libraries.

Here, we demonstrated the generation of triple emulsion picoreactors compatible with FACS sorting. The selection of optimal surfactants was aided by a rapid plate-based screening strategy that identified surfactants compatible with in situ expression of protein candidates for enzyme screening. This plate-based pre-optimization approach for a hydrocarbon/aqueous emulsion is extensible to other organic solvents not immediately compatible with these microfluidic droplets (e.g., fluorocarbon miscible solvents). This approach is also compatible with other droplet-based screening platforms (e.g., FADS). As one example application, we demonstrated that these triple emulsions can encapsulate a fluorogenic lipase reaction in the octanol/aqueous core using a hydrophobic substrate where turnover is limited by transfer kinetics. These results lay the foundation for future lipase engineering for biodiesel production from longer and more hydrophobic esters (e.g., plant oil fatty acids).

Compared to other microfluidic methods, this method uses simple, robust microfluidic devices and commercial sorting machines, enabling screening at very high throughput using minimal amounts of reagents. Even at conservative sorter analysis rates of 100–200 Hz, 1 million droplet reactions can be screened in 2–3 h, making it possible to screen tens of millions of reactions per day. Moreover, the small internal volumes demonstrated here (≈20 pL) make it possible to screen 10^6^ reactions using 20 μL of reagents. Assuming reactions of 5 μL for each phase in 1536-well plates, this results in a 500 000-fold reduction in reagents from 10 L of reagents over 10^6^ reactions in >650 plates. Compared to benchtop scale experiments where the less dense hydrocarbon phase has the tendency to separate or cream, encapsulating biphasic reactions within a droplet picoreactor ensures that the hydrocarbon phase is well dispersed within the aqueous phase without the need for mixing.

Further technological developments may improve the robustness and applicability of triple emulsion picoreactors to diverse applications. For example, redesigning the droplet generator to form octanol/aqueous droplets on-chip upstream of encapsulation could eliminate the need for the pre-emulsification step and result in more regular droplet geometries. Similarly, the addition of multiple inlet channels could enable more complex on-chip mixing, facilitating condition screening. Polymerizable or mineralizable shells may expand the range of compatible organic solvents beyond those that are insoluble in HFE7500 and other fluorocarbon oils.^[52,53]^ In the future, FACS sortable triple emulsion picoreactors could be used to test assay conditions with industrially relevant non-aqueous phases such as PET plastics (polyethylene terephthalate),^[54]^ petroleum products,^[9,10,55]^ pyrolysis oil from thermocracked plastics,^[24,56,57]^ and recycled cooking oils.^[4,58]^ In biphasic reactions with these hydrocarbon phases, catalysts – either small molecule or enzymatic – could be screened from libraries where each DNA-barcoded bead displays many copies of a small molecule, enzyme, or gene.^[59–64]^ With the scale possible from such a platform, the data generated would both complement and enable robust learning algorithms for catalyst design.

## 4. Experimental Section

Extended methods are available in Supporting Information.

### Accessible Data and Materials:

Plasmid constructs are deposited in Addgene under accession numbers 216 849, 226 840, and 226 842. All Python scripts used for data and image analysis are available at https://osf.io/gbq5r/ with all inputs used in the analysis.

### Plate Reader Screening:

Solutions of hydrocarbon solvents (Sigma–Aldrich) and PBS pH 7.4 (Gibco) were pre-mixed with surfactants at concentrations of 0.625-5% (w/v). Hydrocarbon solution (25 μL) was added to 75 μL of aqueous solution in wells of a 96-well plate (Nunc). Plates were vortexed before reading optical absorbance in a multi-mode plate reader (Tecan) at 2 and 24 h. At 24 h, all wells were imaged with an optical microscope (AmScope). Turbidity values were calculated from the absorbance of 400 nm light using custom Python scripts.

### Droplet Generation:

Droplets were generated of custom fabricated PDMS (polydimethylsiloxane) devices using previously reported designs.^[38]^ Input solutions described in Figures 3, 4, and 6 were loaded into plastic syringes (BD) and connected to the PDMS device via LDPE medical tubing (Scientific Commodities Inc.). Syringe pumps drove the flow of reagents into the device, with standard flow rates of 100 μL h^−1^ for each inner solution, 400 μL h^−1^ for the fluorocarbon oil, and 4000 μL h^−1^ for the outer aqueous phase for 45 μm devices. For 30 μm devices, standard flow rates are 30, 110, and 3500 μL h^−1^, respectively. For triple emulsions with PBS as the aqueous phase, inner solutions were generated from hydrocarbon and aqueous solutions used in the plate reader screen. Octanol was labeled with 400 μm Nile red (Sigma–Aldrich). For protein expression in droplets, the two inner solutions were comprised of 1) PURExpress reagents (NEB) or 2) emulsions of octanol + 5% (w/v) Span 80 + 40 μm Nile Red in an aqueous phase of nuclease-free water (Promega) + 200 ng μL^−1^ miniprepped plasmid + 5% (w/v) Tween-20. For lipase turnover in droplets, the two inner solutions were comprised of 1) 1% (w/v) Tween-20 with BsLipA variants attached to Streptavidin-coated Dynabeads (Invitrogen) via a biotinylated anti-GFP antibody (Abcam) or 2) emulsions of octanol + 5% (w/v) Span 80 + 100 μm Resorufin butyrate (Santa Cruz Biotechnology) in an aqueous phase 394 mm HEPES (Sigma) pH 7.4.

### Microscopy:

Droplet emulsions were images with bright field and fluorescence microscopy (Nikon) using green, red-orange, and red fluorescence filter cubes. Flatfield correction, particle detection, and fluorescence quantification were performed using custom Python scripts and the cv2/OpenCV library.

### Fluorescence Activated Cell Sorting:

Droplets were sorted on a FACSAria II cell sorter (BD) with a 130 μm nozzle. Laser delays were manually calibrated with 32 μm AccuCount Ultra Rainbow beads (Spherotech). Forward and side scattering voltage were manually optimized samples of double emulsions. Droplet delays were manually calibrated by sorting 50 double emulsions onto a glass slide at each setting and manually counting the recovered droplets by optical microscopy. Detector voltages were optimized using triple emulsion samples with expressed GFP and Nile Red labeled octanol.

## Supplementary Material

Supporting Information

Supporting Data

Supporting Information is available from the Wiley Online Library or from the author.

## Data Availability

The data that support the findings of this study are openly available in Open Science Framework (OSF) at 10.17605/OSF.IO/GBQ5R, reference number gbq5r.
